# Complete mitochondrial genome sequence of the Indian clouded leopard (*Neofelis nebulosa*)

**DOI:** 10.1080/23802359.2016.1214543

**Published:** 2016-09-05

**Authors:** Wajeeda Tabasum, Ara Sreenivas, Kesav Kumar Bheemavarapu, Tirupathi Rao Golla, Ajay Gaur

**Affiliations:** Conservation Genetics Lab, LaCONES, CSIR-Centre for Cellular and Molecular Biology, Hyderabad, India

**Keywords:** Clouded leopard, complete mitochondrial genome, ngs, conservation

## Abstract

The complete mitochondrial genome of sequence 16,859 bp of Indian clouded leopard (*Neofelis nebulosa*) has been sequenced using next generation sequencing technology Torrent ^PGM^ platform. The complete mitochondrial genome sequence of clouded leopard consists of 13 protein-coding, 22 tRNA, and two rRNA genes and a control region (CR). The mitochondrial genome is relatively similar to other felid mitochondrial genomes with respect to gene arrangement, composition, tRNA structures and skews of AT/GC bases to be typical of those reported for other mammals. The nucleotide composition of the genome shows that there is more A–T% than G–C% on the positive strand as revealed by positive AT and CG skews. The base composition of the mitochondrial genome of clouded leopard is as follows: A, 5362 bp (31.8%); C, 4560bp (27.0%); G, 2475 bp (14.6%); T, 4462 bp (26.4%). Most of the genes have ATG initiation codons, except ND1, ND2, ND3, ND4, ND6, and CYTB (ATA start codon).

The clouded leopard (*Neofelis nebulosa*) is a member of sub-family Pantherinae, which includes six big cats, namely, tiger, lion, leopard, snow leopard, clouded leopard and jaguar (Johnson et al. 1996, 2006). Clouded leopard has recently been split into two species. *Neofelis nebulosa* is restricted to mainland Southeast Asia and *Neofelis diardi* is found on the islands of Sumatra and Borneo (Buckley-Beason et al. 2006; Kitchener et al. 2006; Wilting et al. 2007). The overall estimated population is less than 10,000 mature individuals in the wild, with a declining quality of habitat and exploitation (Nowell & Jackson 1996).

The blood sample used for DNA extraction and analysis was collected from a female clouded leopard named Rehana with a studbook number 00018 from Sepahijala Zoological Park, Agartala, India (N: 230 40’22.39”, E: 910 19’13.20”).

In this study, the complete mitochondrial genome of clouded leopard was sequenced and characterized. Two sets of primers were designed based on highly conserved sequences of an alignment with full-length mitochondrial genomes from the available public database and used for PCR, with an average amplicon size of 8488 bp each with an overlap of 1177 bp. The characteristics of mitochondrial genome of clouded leopard were identical to the typical vertebrate mitochondrial genome. The complete mitogenome of clouded leopard is 16,859 bp in length (GeneBank accession number KU133958), which is made up of 37 genes, which includes 13 protein-coding genes, two rRNA genes, and 22 tRNA genes, as well as a control region, is highly conserved among most vertebrates mitochondrial genome (Boore 1999).

Gene order and origin of reading frame of all protein coding genes were identical to other members of Carnivora. Except for ND6 and nine tRNA genes, all of the other mitochondrial genes are encoded on the heavy strand. The total length of the 13 protein-coding genes was 11,398 bp, which corresponds to 67.60% of the mitochondrial genome sequence length. The longest gene was ND5 (1806 bp) and the shortest gene was ATP8 (198 bp). The start and termination codons appeared universal among all species. Except for ND1, ND2, ND3, ND4 ND6 and CYTB (ATA start codon), the remaining protein-coding genes start with ATG. In clouded leopard, origin of L-strand replication (OL) was within a cluster of five tRNA genes i.e.: tRNA^Trp^, tRNA^Ala^, tRNA^Asn^, tRNA^Cys^ and tRNA^Tyr^. The noncoding region (the CR) was located between the tRNA-Pro and tRNA-Phe genes and is 1411 bp.

The phylogenetic position of clouded leopard was estimated using maximum-likelihood (ML) and Bayesian inference (BI) (Figure 1) containing concatenated 13 protein-coding genes of 14 species derived from different families in sub-order feliformia. Bayesian analysis was performed using MRBAYES v 3.1.2 (Huelsenbeck et al. 2001) with four chains of 1.1 × 10^5^ generations and sampling the trees every 100 generations. ML tree was similar to the BI tree. The monophyly of genus *panthera* was clearly depicted and were statistically supported by high bootstrap values. This characterization of clouded leopard mitogenome will contribute to more refined phylogeny of big cats and also facilitate concentrated efforts towards the conservation of this species.

**Figure 1.**
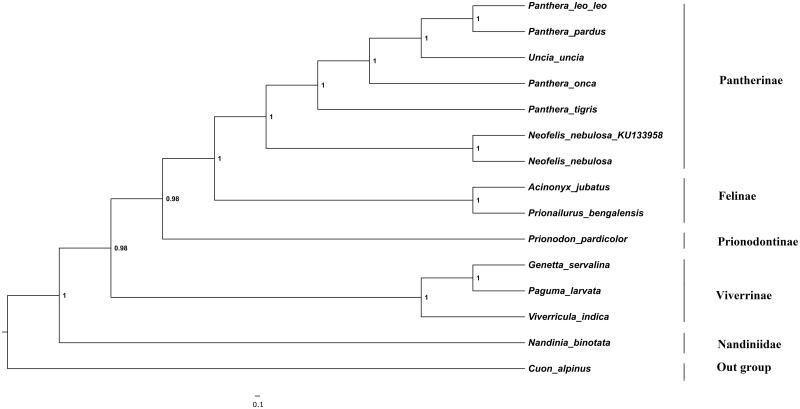
The Bayesian majority-rule consensus tree is inferred from the combined data set of 13 mitochondrial protein-coding genes. The node value represents the Bayesian posterior probabilities. The phylogenetic tree was rooted using *Cuon alpines* (NC_013445). Our sample sequence was *Neofelis nebulosa* (KU_133958). The analyzed species and corresponding NCBI accession number as follows: *Panthera leo leo* (KF776494), *Panthera pardus* (EF_551002), *Uncia uncia* (NC_010638), *Panthera onca* (KM_236783), *Panthera tigris* (EF_551003), *Neofelis nebulosa* (NC_008450), *Acinonyx jubatus* (NC_005212), *Prionailurus bengalensis* (KP_246843), *Nandinia binotata* (NC_024567), *Viverricula indica* (NC_025296), *Genetta servalina* (NC_024568), *Paguma larvata* (NC_029403) and *Prionodon pardicolor* (NC_024569).

## Nucleotide sequence accession number

The complete genome sequence of Indian Clouded leopard (*Neofelis nebulosa*) has been assigned Genbank accession number KU133958.

## References

[CIT0001] Boore JL. 1999 Animal mitochondrial genomes. Nucleic Acids Res. 27:1767–1780. 10101183 10.1093/nar/27.8.1767 PMC148383

[CIT0002] Buckley-Beason VA, Johnson WE, Nash WG, Stanyon R, Menninger JC, Driscoll CA, Howard J, Bush M, Page JE, Roelke ME, et al 2006 Molecular evidence for species level distinctions in clouded leopards. Curr Biol. 16:2371–2376. 17141620 10.1016/j.cub.2006.08.066 PMC5618441

[CIT0003] Huelsenbeck JP, Ronquist F, Nielsen R, Bollback JP. 2001 Bayesian inference of phylogeny and its impact on evolutionary biology. Science. 294:2310–2314. 11743192 10.1126/science.1065889

[CIT0004] Johnson WE, Dratch PA, Martenson JS, O’Brien SJ. 1996 Resolution of recent radiations within three evolutionary lineages of Felidae using mitochondrial restriction fragment length polymorphism variation. J Mamm Evol. 3:97–120.

[CIT0005] Johnson WE, Eizirik E, Pecon-Slattery J, Murphy WJ, Antunes A, Teeling E, O’Brien SJ. 2006 The late Miocene radiation of modern Felidae: a genetic assessment. Science. 311:73–77. 16400146 10.1126/science.1122277

[CIT0006] Kitchener AC, Beaumont MA, Richardson D. 2006 Geographical variation in the clouded leopard, *Neofelis nebulosa*, reveals two species. Curr Biol. 16:2377–2383. 17141621 10.1016/j.cub.2006.10.066

[CIT0007] Nowell K, Jackson P. 1996 Wild cats. Status survey and conservation action plan. Gland, Switzerland: IUCN.

[CIT0008] Wilting A, Buckley-Beason VA, Feldhaar H, Gadau J, O’Brien SJ, Linsenmair KE. 2007 Clouded leopard phylogeny revisited: support for species recognition and population division between Borneo and Sumatra. Front Zool. 4:15. 17535420 10.1186/1742-9994-4-15 PMC1904214

